# Pleomorphic lobular carcinoma of the male breast with axillary lymph node involvement: a case report and review of literature

**DOI:** 10.1186/1472-6890-14-16

**Published:** 2014-04-27

**Authors:** Muhammad Nauman Zahir, Khurram Minhas, Munira Shabbir-Moosajee

**Affiliations:** 1 Department of Oncology, Aga Khan University Hospital, Stadium Road, PO BOX: 3500, Karachi 74800, Pakistan; 2 Department of Pathology and Microbiology, Aga Khan University, Stadium Road, PO BOX: 3500, Karachi 74800, Pakistan

**Keywords:** Male breast cancer, Pleomorphic lobular carcinoma, E-cadherin

## Abstract

**Background:**

Carcinoma of the male breast is responsible for less than 1% of all malignancies in men but the incidence is rising. Invasive ductal carcinoma is the most common histological subtype while invasive lobular carcinoma is responsible for only 1.5% of the total cases of which pleomorpic lobular carcinoma is an extremely rare variant. We report the case of a gentleman with node positive, pleomorphic lobular carcinoma of the breast.

**Case presentation:**

An elderly gentleman with a past history of type 2 diabetes and long term ethanol use presented to us with a self-discovered palpable lump in the left breast. Physical examination revealed bilateral gynaecomastia along with a well circumscribed subareolar mass and palpable lymphadenopathy in the ipsilateral axilla. The breast nodule revealed atypical cells on fine needle aspiration biopsy and the patient underwent a modified radical mastectomy after systemic surveillance was negative for metastatic disease. The lesion was reported as grade III pleomorphic lobular carcinoma with a lack of E-cadherin expression on immunohistochemistry and the neoplastic cells exhibited strong positivity for estrogen receptor in the absence of Her2 gene amplification. Six out of the eleven dissected regional lymph nodes showed evidence of disease. The patient completed 4 cycles of adjuvant chemotherapy without evidence of recurrent disease and was subsequently lost to follow up.

**Conclusions:**

Although invasive lobular carcinomas comprise 12% of all female breast cancers, they are very rare in males due to lack of acini and lobules in the normal male breast. Pleomorphic lobular carcinoma, an aggressive variant of ILC is even rarer in males.

Chronic consumption of ethanol by our patient may have resulted in some degree of hepatic impairment with resultant hyperestrogenism. This in theory may have been the cause of his gynaecomastia, resultant breast cancer and is a plausible explanation for development of the invasive lobular subtype in a male. The prognosis and clinicopatholocial features of pleomorphic lobular carcinoma in men are less clearly defined due to its rarity. Additional studies are hence necessary to improve our understanding of this disease in males.

## Background

Carcinoma of the male breast is a rare entity accounting for 0.7% of all breast cancers [1]. Although the disease is also responsible for less than 1% of all malignancies in men, the incidence of male breast cancer has seen a rise of about 26% over the past quarter of a century [1,2]. Risk factors similar to those observed for female breast cancers are responsible for pathogenesis. All histopathological variants seen in female breast cancer have been observed [3,4]. Invasive ductal carcinoma is the most common histological subtype accounting for approximately 85% of all cases. Male breast cancers are significantly more likely to exhibit hormone receptor expression in comparison to female breast cancers [1,2,5]. Data from the Surveillance, Epidemiology, and End Results (SEER) database shows that only 1.5% of male breast cancers are of the invasive lobular subtype while the same histological variant is responsible for approximately 12% of carcinomas of the female breast [2].

Herein we report the case of an elderly gentleman diagnosed with the pleomorphic variant of invasive lobular carcinoma along with involvement of the ipsilateral axillary lymph nodes. Only three cases of this histopathological subtype have ever been reported in the male breast and none thus far had shown metastasis to the regional draining lymph nodes.

## Case presentation

A 68 year old Pakistani male was referred to the outpatient oncology clinic at our center with a three month history of a self-discovered, progressively increasing palpable lump in the left breast. He had sought medical attention for increasing size of the lump with recent development of pain. His past history was significant for type 2 diabetes and long term ethanol use. There was no family history of breast disease.

Physical examination revealed bilateral gynaecomastia along with a 3 × 3 cm sized, relatively well circumscribed subareolar mass which was firm and mildly tender. The overlying skin appeared normal. There was evidence of palpable lymphadenopathy in the ipsilateral axilla.

**Figure 1:**
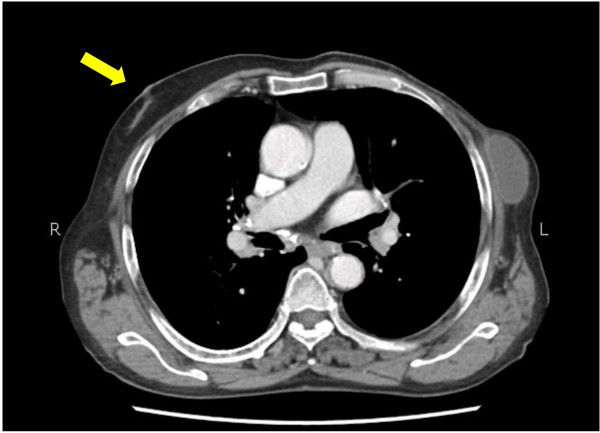
Computed tomographic image highlighting right sided gynaecomastia (yellow arrow) and left sided postoperative changes with seroma formation.

Breast ultrasonogram confirmed bilateral gynaecomastia along with an irregular, hypoechoic, non-compressible mass in the subareolar region of the left breast with associated ipsilateral axillary adenopathy. The breast nodule was subjected to fine needle aspiration biopsy (FNAB) which revealed atypical cells, following which the patient underwent baseline blood workup and complete staging with computed tomographic (CT) imaging. His preoperative blood workup was essentially normal although liver function tests did reveal a marginally elevated alanine transaminase (ALT) at 64 IU/L. Serological tests for hepatitis B and C were negative and ultrasound of the liver showed mild fatty infiltration. After a core biopsy confirmation of malignancy, he underwent a modified radical mastectomy (MRM) with axillary lymph node dissection. His postoperative course was uneventful except mild seroma formation at the surgical site (Figure 1).

**Figure 2:**
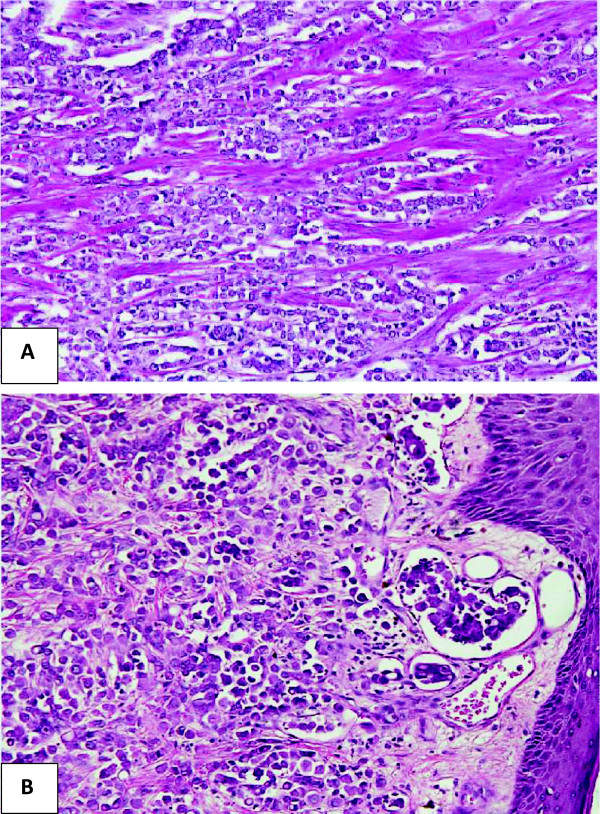
**Photomicrographs of the tumor cells.** Magnification; ×40: Tumor cells arranged in aggregates and as cords **(A)**. Magnification; × 40: Tumor cells exhibiting pleomorphic cells along with a focus of dermal lymphatic invasion **(B)**.

Gross histopathology of the surgical specimen revealed a firm greyish white vaguely circumscribed lesion just beneath the nipple measuring 2.8 × 2.5 × 2 cm. Microscopically, an infiltrative tumor was identified which was composed of discohesive sheets of pleomorphic cells. Tumor cells showed moderate to marked nuclear pleomorphism along with moderate cytoplasm (Figure 2). There was absence of an intraductal component whereas gynaecomastia was evident in the surrounding breast tissue characterized by breast ducts lined by double layer of epithelium with mild ductal hyperplasia. Breast acini or lobules were not identified. The lesion was reported as grade III on a scale of I to III according to modified Bloom Richardson grading system with invasion of the dermal lymphatics. Surgical margins were negative and six of the eleven dissected axillary lymph nodes demonstrated evidence of disease. The tumor was pathologically staged as pT2pN2Mx.

**Figure 3:**
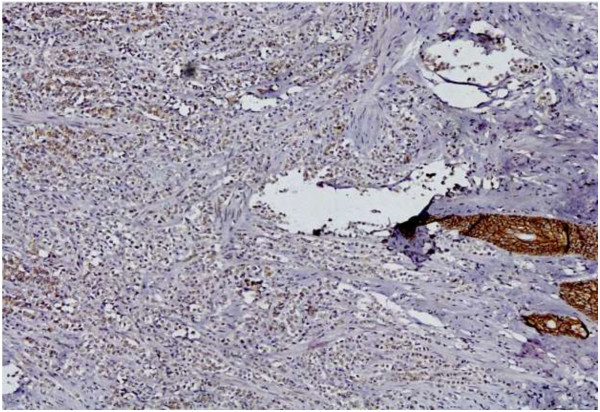
Magnification; × 20: Negative E-cadherin immunostaining of tumor cells with positive internal control in skin adnexal structures.

Tumor cells were negative for E-cadherin immunostain (Figure 3) and thus based on morphology and immunprofile a diagnosis of invasive lobular carcinoma, pleomorphic variant was rendered. The neoplastic cells were strongly positive for estrogen receptor expression but only displayed weak positivity for expression of the progesterone receptor. Her2-Neu staining by immunohistochemistry revealed a score of +1 and the Ki-67 proliferative index was 20-25%.

The patient was recommended adjuvant chemotherapy to be followed by chest wall and axillary irradiation and hormonal therapy. He completed 4 cycles of adjuvant chemotherapy without any evidence of recurrence and was subsequently lost to followup.

## Conclusions

Although the majority of men with breast cancer have no identifiable risk factors, some risk factors that are modestly unique to men include never being married, gynecomastia, Klinefelter’s syndrome and a history of testicular or liver pathology [6].

Breast cancers in men typically presents as a painless, firm mass that is usually subareolar. The left breast is involved slightly more often than the right, and less than one percent of cases are bilateral [7,8]. Male breast cancers tend to present sooner because of scarcity of breast tissue and hence most subjects have early stage disease at presentation.

Invasive lobular carcinoma (ILC) of the breast is a histopathologically distinct entity from the much more common invasive ductal carcinoma (IDC). Although invasive lobular carcinomas comprise 12% of all female breast cancers, they are very rare in the male breast [1]. The rarity of a lobular histologic subtype of breast cancer is due to lack of acini and lobules in the normal male breast [9]. Endogenous or exogenous estrogenic stimulation may induce the development of acini and lobules in the male breast and hence subsequently increases the theoretical risk of development of invasive lobular carcinoma (ILC). ILC typically lacks E-cadherin expression on immunohistochemical (IHC) staining and negative expression is now used as an aid in diagnosis [10].

Pleomorphic lobular carcinoma is an aggressive variant of ILC with distinct histopathological features including marked pleomorphism and atypia along with a higher proliferative index [11]. A higher incidence of HER2 gene amplification and a lower frequency of hormone receptor expression are other features typical of the pleomorphic variant when compared to its invasive lobular counterpart [4]. This subtype was first described in the female breast in 1992 by Eusebi et al. who also highlighted the aggressive clinical course associated with it [12]. Only 3 cases of this variant have been reported in the male breast previously [4,10,13] but our case is unique in that it is the first with evidence of regional draining lymph nodal involvement. The clinical and histopathological features of the 4 cases of pleomorphic lobular carcinoma of the male breast described so far, have been summarized in Table 1.

**Table 1:** Clinical and histopathological features of pleomorphic lobular carcinoma of the male breast

	**Maly et al.**	**Rohini et al.**	**Ishida et al.**	**Present case**
**Reference**	10	4	13	
**Year of report**	2005	2010	2013	2013
**Age**	44	55	76	68
**Site**	Left	Left	Right	Left
**Gynecomastia**	No	No	Yes	Yes
**Size (cm)**	2.5 × 2.0	3.0 × 2.5	3.0 × 2.5	2.8 × 2.5
**Estrogen receptor expression**	+	Not mentioned	+	+
**Progesterone receptor expression**	+	Not mentioned	-	+
**Her2 gene amplification**	-	Not mentioned	-	-
**Ki-67 proliferative index (%)**	5	Not mentioned	12.7	20-25
**E-cadherin immunostaining**	-	-	-	-
**Lymph node involvement**	-	-	-	+
**Followup**	Disease free at 2 years	Disease free at 1 year	Disease free at 2 months	Disease free at 3.5 months

The present case merits discussion on several key points. Ethanol use by this gentleman may have resulted in some degree of hepatic impairment. It is an established fact that liver dysfunction results in hyperestrogenism which may have been the cause of his gynaecomastia and resultant breast cancer [14]. The hyperestrogenism theory also explains the development of the invasive lobular subtype in this particular gentleman. The typical morphologic features of pleomorphic lobular carcinoma and lack of E-cadherin expression aided in this rare histopathological diagnosis. The clinical presentation, hormone receptor expression and the absence of Her2 gene amplification was typical of what is seen in male breast cancer. To the best of our knowledge, this is the first reported case of pleomorpic lobular carcinoma in the male breast with regional lymph node involvement. This may be attributed to a delay on the part of the patient to seek appropriate medical care in a resource poor country like Pakistan where all medical expenses are incurred by patients themselves.

ILC and particularly pleomorphic lobular carcinoma in the male breast is a rare occurrence and reminds us to tailor management according to subtype as this variant is known to be clinically aggressive in females. The prognosis and clinicopatholocial features of this variant in men are less clearly defined due to its rarity. Additional studies are hence necessary to improve our understanding of this disease in males.

## Consent

Written informed consent was obtained from the patient for publication of this case report and any accompanying images. A copy of the written consent is available for review by the Editor-in-Chief of this journal.

## Competing interests

The authors declare that they have no competing interests.

## Authors’ contributions

MNZ did the literature search and drafted the manuscript. MSM conceived the case report and helped in drafting the manuscript. KM helped draft key areas of the manuscript in addition to providing the histopathological images. All authors read and approved the final manuscript.

## Pre-publication history

The pre-publication history for this paper can be accessed here:

http://www.biomedcentral.com/1472-6890/14/16/prepub
